# Data on heavy metals and selected anions in the Persian popular herbal distillates

**DOI:** 10.1016/j.dib.2016.05.005

**Published:** 2016-05-12

**Authors:** Mozhgan Keshtkar, Sina Dobaradaran, Farshid Soleimani, Vahid Noroozi Karbasdehi, Mohammad Javad Mohammadi, Roghayeh Mirahmadi, Fatemeh Faraji Ghasemi

**Affiliations:** a Department of Environmental Health Engineering, Faculty of Health, Bushehr University of Medical Sciences, Bushehr, Iran; b The Persian Gulf Marine Biotechnology Research Center, The Persian Gulf Biomedical Sciences Research Institute, Bushehr University of Medical Sciences, Boostan 19 Alley, Imam Khomeini Street, Bushehr, Iran; c Systems Environmental Health, Oil, Gas and Energy Research Center, The Persian Gulf Biomedical Sciences Research Institute, Bushehr University of Medical Sciences, Bushehr, Iran; d Department of Environmental Health Engineering, School of Public Health and Environmental Technologies Research Centre, Ahvaz Jundishapur University of Medical Sciences, Iran

**Keywords:** Daily intake, Herbal distillates, Heavy metals, Selected anions

## Abstract

In this data article, we determined the concentration levels of heavy metals including Pb, Co, Cd, Mn, Mg, Fe and Cu as well as selected anions including ${\mathrm{NO}}_{3}^{-}$ , ${\mathrm{NO}}_{2}^{-}$, ${\mathrm{PO}}_{4}^{-3}$ and ${\mathrm{SO}}_{4}^{-2}$ in the most used and popular herbal distillates in Iran. It is well known that heavy metals may pose a serious health hazard due to their bioaccumulation throughout the trophic chain (“Heavy metals (Cd, Cu, Ni and Pb) content in two fish species of Persian Gulf in Bushehr Port, Iran” (Dobaradaran et al., 2013) [1]; “Comparative investigation of heavy metal, trace, and macro element contents in commercially valuable fish species harvested off from the Persian Gulf” (Abadi et al., 2015) [2]) as well as some other environmental pollutions, “Assessment of sediment quality based on acid-volatile sulfide and simultaneously extracted metals in heavily industrialized area of Asaluyeh, Persian Gulf: concentrations, spatial distributions, and sediment bioavailability/toxicity” (Arfaeinia et al., 2016) [3]. The concentration levels of heavy metals and anions in herbal distillates samples were determined using flame atomic absorption spectrometry (FAAS, Varian AA240, Australia) and a spectrophotometer (M501 Single Beam Scanning UV/VIS, UK) respectively.

**Specifications Table**

**Table:** 

Subject area	Chemistry
More specific subject area	Plant Medicine
Type of data	Table
How data was acquired	Flame Atomic Absorption Spectrometry (FAAS, Varian AA240, Australia) and a spectrophotometer (M501 Single Beam Scanning UV/VIS, UK)
Data format	Raw, analyzed
Experimental factors	All herbal distillates bottles were stored in a dark place at room temperature in their original sealed plastic containers until the analysis.
Experimental features	Determine the concentration levels of heavy metals including Pb, Co, Cd, Mn, Mg, Fe and Cu as well as selected anions including ${\mathrm{NO}}_{3}^{-}$ , ${\mathrm{NO}}_{2}^{-}$, ${\mathrm{PO}}_{4}^{-3}$ and ${\mathrm{SO}}_{4}^{-2}$
Data source location	Bushehr, Iran
Data accessibility	Data is with this article.

**Value of the data**

• Data can be used as a base-line data for concentration levels of some metals in herbal distillates.
• Data shown here may motivate further studies on medical benefits of herbal distillates due to their low concentration levels of metals.
• Data shown here may serve as benchmarks for other groups working in the field of pharmacology and toxicology.

## 1 Data

In the data, characteristics of plants used for extracting herbal distillates are presented in Table 1. The data in Table 2 show that Pb, Cd, Mn, and Cu were not detected (ND) in all examined herbal distillate samples, but the mean concentration levels of Co, Mg, and Fe were 6.5, 1163.7, and 91 with a range of ND-30, 90-8970, and ND-500 μg/l respectively, and the mean concentration levels of ${\mathrm{NO}}_{3}^{-}$, ${\mathrm{NO}}_{2}^{-}$, ${\mathrm{PO}}_{4}^{-3}$ and ${\mathrm{SO}}_{4}^{-2}$ were 527.5, 17.5, 43, and 24 μg/l respectively. The data in Table 3 show that the maximum daily intakes of Co, Mg and Fe reached 3, 897 and 50 µg/day respectively based on 100 ml daily use by local consumer. It should be noted as other metals including Pb, Cd, Mn and Cu were not detected in all analyzed samples, daily intakes for these metals were not calculated.

## 2 Experimental design, materials and methods

Twenty different herbal distillates of the most used and popular herbal distillates were purchased from herbal distillate distribution shops in Bushehr, Iran. All purchased herbal distillates were produced by traditional methods from Ordibehesht Company in Meymand city, which is the main city in Iran for herbal distillates production. All herbal distillates bottles were stored in a dark place at room temperature in their original sealed plastic containers until the analysis. Before taken for analysis, samples were collected in 100 ml sterile glass bottles that were previously washed and dried in oven at 180 °C. The concentration levels of heavy metals and anions in herbal distillates samples were determined using flame atomic absorption spectrometry (FAAS, Varian AA240, Australia) and a spectrophotometer (M501 Single Beam Scanning UV/VIS, UK) respectively. The limit of detection (LOD) was calculated as $\;3S_{b}/b$, where $S_{b}$ is the standard deviation for twenty measurements of the calibration blank, and b is the slope of the calibration curve.

## Figures and Tables

**Table 1:** Characteristics of plants used for extracting herbal distillates [4], [5], [6], [7].

**Common name**	**Scientific name**	**Claimed therapeutic property**
Alfalfa	Medicago sativa	Fattening, slimming treatment, blood purification
Aloe vera	Aloe vera	Power amplifier, blood purifier
Camelthorn	Alhagi maurorum	Blood purifier, kidney detersive
Chicory	Cichorium intybus	Treatment of liver and gallbladder disorder, curing constipation
Dog-rose	Rosa canina	Carminative, skin care
Fennel	Foeniculum vulgare	Antiseptic, palliative and anti-inflammatory effects
Fenugreek	Trigonella foenum-graecum	Digestive problems and antidiabetic
Fumitory	Fumaria officinalis	Bile disorders, eye irritation
Herbal mixture	–	Sedative, used for upset stomach
Lavender	Lavandula stoechas	Amplifier neurology soothing, anticonvulsants
Licorice	Glycyrrhiza glabra	Impact on the digestive system, Treating swelling and ulcers
Nettle	Urtica	Treatment of respiratory, anti-diarrhea, anti-inflammatory
Olive	Olea europaea	Disposal of gallstones, appetizer
Orange blossom	Citrus sinensis	Invigorating for the skin, relaxing effect on mind and body
Pussy willow	Salix aegyptiaca	Mild sedative, skin care
Reppermint	Mentha	Improved upset stomach and indigestion, skin irritation
Rose	Rosa damascena	Mild sedative, skin treatments
Sycamore	Platanus orientalis	Improving blood circulation to brains and heart
Walnut	Juglans regia	Anti-diarrhea, hypoglycemia
Yarrow	Achillea	Anticonvulsants, febrifuge

**Table 2:** Concentration levels of heavy metals, selected anions and regulatory limit values (μg/l) in the analyzed herbal distillates.

***n*=20**	**Pb**	**Co**	**Cd**	**Mn**	**Mg**	**Fe**	**Cu**	$NO_{3}^{-}$	$NO_{2}^{-}$	$PO_{4}^{-3}$	$SO_{4}^{-2}$
**Detection limit**	**0.01**	**0.005**	**0.002**	**0.002**	**0.002**	**0.006**	**0.003**	–	–	–	–
Fenugreek	^⁎^ND	ND	ND	ND	300	60	ND	510	20	90	20
Walnut	ND	ND	ND	ND	90	ND	ND	570	20	50	20
Alfalfa	ND	ND	ND	ND	90	60	ND	380	20	30	30
Yarrow	ND	ND	ND	ND	700	60	ND	540	10	40	10
Lavender	ND	ND	ND	ND	380	20	ND	610	20	30	30
Fennel	ND	ND	ND	ND	580	ND	ND	480	20	50	20
Sycamore	ND	ND	ND	ND	370	70	ND	550	10	20	30
Aloe vera	ND	ND	ND	ND	320	100	ND	710	20	30	ND
Olive	ND	ND	ND	ND	95	ND	ND	570	20	30	30
Nettle	ND	ND	ND	ND	300	50	ND	640	20	30	40
Licorice	ND	10	ND	ND	200	ND	ND	600	20	20	20
Orange blossom	ND	20	ND	ND	1300	ND	ND	470	10	20	40
Fumitory	ND	ND	ND	ND	520	500	ND	370	10	40	40
Dog-rose	ND	10	ND	ND	1980	ND	ND	550	20	70	70
Camelthorn	ND	10	ND	ND	390	ND	ND	540	10	30	20
Reppermint	ND	10	ND	ND	8970	200	ND	570	30	40	30
Pussy willow	ND	30	ND	ND	460	500	ND	560	10	40	10
Chicory	ND	10	ND	ND	830	200	ND	460	20	80	ND
Rose	ND	20	ND	ND	5110	ND	ND	310	20	20	ND
Herbal mixture	ND	10	ND	ND	290	ND	ND	560	20	100	20
Minimum value	–	ND	–	–	90	ND	–	310	10	20	ND
Maximum value	–	30	–	–	8970	500	–	710	30	100	70
Mean value	–	6.5	–	–	1163.7	91	–	527.5	17.5	43	24
Std. deviation	–	8.75	–	–	2154	153	–	92.52	5.36	20.32	16.24
JECFA^a^ (μg /l-bwt)	25	–	7	–	–	–	50				
ATSDR^b^ (μg/l-day)	–	0.01	0.0001	–	–	–	0.01				

^⁎^None Detect.

a Joint FAO/WHO Expert Committee on Food Additives.

b US Agency for Toxic Substances and Disease.

**Table 3:** The estimated daily intakes of heavy metals for the herbal distillate samples.

**Herbal distillates**	**Co**	**Mg**	**Fe**
Fenugreek	–	30	6
Walnut	–	9	–
Alfalfa	–	9	6
Yarrow	–	70	6
Lavender	–	38	2
Fennel	–	58	–
Sycamore	–	37	7
Aloe vera	–	32	10
Olive	–	9.5	–
Nettle	–	30	5
Licorice	1	20	–
Orange blossom	2	130	–
Fumitory	–	52	50
Dog-rose	1	198	–
Camelthorn	1	39	–
Reppermint	1	897	20
Pussy willow	3	46	50
Chicory	1	83	20
Rose	2	511	–
Herbal mixture	1	29	–
Minimum value	1	9	2
Maximum value	3	897	50
Mean value	0.65	116.37	9.1
Std. deviation	0.88	215.16	15.27
